# Synergistic antifungal effects and mechanisms of amantadine hydrochloride combined with azole antifungal drugs on drug-resistant *Candida albicans*

**DOI:** 10.3389/fcimb.2025.1455123

**Published:** 2025-02-26

**Authors:** Xiuyun Li, Yuanyuan Zhi, Ximeng Duan, Xu Chen, Min Cui, Shicun Zheng

**Affiliations:** ^1^ Infection and Microbiology Research Laboratory for Women and Children, Shandong Provincial Maternal and Child Health Care Hospital Affiliated to Qingdao University, Jinan, Shandong, China; ^2^ Gynecology Department, Shandong Provincial Maternal and Child Health Care Hospital Affiliated to Qingdao University, Jinan, Shandong, China; ^3^ Pharmacy Department, Taizhou Central Hospital (Taizhou University Hospital), Taizhou, Zhejiang, China

**Keywords:** amantadine hydrochloride, fluconazole, drug-resistant *Candida albicans*, drug combination, *Galleria mellonella*

## Abstract

**Introduction:**

The increasing resistance of *Candida albicans* (*C. albicans*) to conventional antifungal drugs poses a great challenge to the clinical treatment of infections caused by this yeast. Drug combinations are a potential therapeutic approach to overcome the drug- resistance of *C. albicans*. This study explored the synergistic effects of amantadine hydrochloride (AMH) combined with azole antifungal drugs against drug-resistant *C. albicans in vitro* and *in vivo*.

**Methods:**

The *in vitro* sensitivity of *Candida* spp. to drugs was determined by the microdilution method. The effect of drugs on the efflux pump activity of *C. albicans* was determined by the rhodamine 6G tracer method. The egg yolk agar plate method was used to determine the activity of extracellular phospholipase, a *C. albicans* virulence factor. The *Galleria mellonella* model of *C. albicans* infection was used to test the *in vivo* efficacy of the combination therapy.

**Results:**

*In vitro* experiments showed that combinations of AMH with azole antifungal drugs had synergistic antifungal effects on planktonic cells of drug-resistant *C. albicans*, with fractional inhibitory concentration index values of <0.5. The *in vivo* synergistic effects and mechanism of drug combinations with AMH were further studied using fluconazole (FLC) as a representative azole antifungal drug. *In vivo*, *G. mellonella* larvae were used to evaluate the antifungal efficacy of AMH +FLC. AMH + FLC treatment increased the survival rate of larvae infected with drug-resistant *C. albicans* and reduced tissue invasion. Studies of the mechanism of synergy showed that AMH inhibited drug efflux pump activity in drug-resistant *C. albicans*, and that AMH + FLC synergistically inhibited early biofilms and the extracellular phospholipase activity of drug-resistant *C. albicans*.

**Conclusion:**

This study provides strong evidence that combinations of non-antifungal drugs and antifungal drugs can effectively overcome drug-resistant *C. albicans* infection. Both AMH and FLC are FDA-approved drugs, eliminating concerns about safety. Our findings provide a foundation for further clinical antifungal research.

## 1 Introduction

Fungal infections have become a significant public health concern, affecting more than 1.5 billion individuals worldwide each year (Brown et al., 2012). Most fungal infections are superficial, but invasive infections can be life-threatening, especially in immunocompromised individuals, such as those with host barrier rupture, neutropenia, cancer or acquired immunodeficiency syndrome. These conditions are all high-risk factors for fungal infection (Almeida et al., 2019; Ma et al., 2021). Azoles are a class of antifungal drugs with optimal selectivity and safety profiles. These fungistatic agents act by inhibiting cytochrome P-450DM (Como and Dismukes, 1994). Fluconazole is a representative azole with a broad antifungal spectrum, low hepatotoxicity, good oral absorption, high bioavailability and extensive tissue distribution. However, the emergence and spread of resistant fungal species are gradually eroding the utility of the current repertoire of azoles used to treat invasive infections (Arastehfar et al., 2020). The small number of antifungal targets unique to fungi and the tolerance and resistance of fungi to existing antifungal drugs make the development of new antifungal drug treatment strategies an urgent task. One potential strategy for overcoming antifungal resistance and recurrenceis to make existing antifungal drugs more effective, such as through chemical optimization (Malik et al., 2012; Wu et al., 2014). Another approach is to identify compounds that sensitize resistant fungi to antifungal drugs. These sensitizers can act in various ways, such as inhibiting efflux pump expression or the fungal stress response (Li et al., 2023c).

Among pathogenic fungi, *Candida* species are especially concerning because they are a leading cause of systemic fungal infections with mortality rates reaching 50% (Kett et al., 2011; Pappas et al., 2018). Moreover, the antifungal resistance of *Candida* species can-not be ignored. In its 2019 Antibiotic Resistance Threats in the United States report, the Centers for Disease Control and Prevention (CDC) classified drug-resistant *Candida* as a serious threat, with an estimate of 34,800 cases and 1,700 deaths (CDC, 2019). The CDC noted that approximately 7% of all *Candida* isolates from blood samples are resistant to FLC.

*Candida albicans* is the most common yeast species of *Candida*, and resistance of *C. albicans* to antifungals is a severe problem (Yu et al., 2022). We previously explored the treatment of drug-resistant *C. albicans*, with combinations of non-antifungal drugs and antifungal drugs, such as ambroxol + FLC, and eravacycline +FLC, among others (Li et al., 2017, 2023b). Ribavirin and FLC were reported to have synergistic effects against drug-resistant *C. albicans* (Zhang et al., 2020). Based on this finding, we explored the potential effects of other combinations of antiviral drugs and azole antifungal drugs against drug-resistant *C. albicans*. Amantadine hydrochloride (AMH) was the first antiviral drug used to suppress the influenza virus. The United States approved AMH as a preventive drug for influenza in 1966, and further confirmed it as a therapeutic drug for influenza in 1976. The efficacy and safety of AMH in adult patients are widely recognized (Jefferson et al., 2000). AMH was later found to have mild anti-Parkinson’s activity, and is now used mainly to treat Parkinson’s disease (Crosby et al., 2003).

The aims of this study were to evaluate the antifungal activity of the combination of AMH and FLC against drug-resistant *C. albicans in vitro* and *in vivo*, and to explore the potential synergistic mechanisms. This study expands our understanding of the clinical application of AMH as a sensitizer of antifungal drugs, and suggests new strategies for combatting drug-resistant fungal infections from a combination drug perspective.

## 2 Materials and methods

### 2.1 Strains and agents

Clinical isolates of three *Candida* species, namely, *C. albicans*, *C. glabrata*, and *C. krusei*, were kindly provided by Professor Sun (Qianfoshan Hospital, Jinan, China). *C. albicans* strains CA4 and CA8, *C. glabrata* strains CG1 and CG8, and *C. krusei* strains CK2 and CK3 are susceptible to azole antifungals; *C. albicans* strains CA10 and CA16, *C. glabrata* strains CG2 and CG3, and *C. krusei* strains CK9 and CK10 are resistant to azoles antifungals, and are designated as drug-resistant strains (CLSI, 2020). All strains were grown on YPD (yeast extract-peptone-dextrose) agar medium. Before each experiment, fungal cells were propagated in YPD medium at 35°C overnight with rotation at 200 rpm. Because AMH+FLC had the best synergistic effect against *C. albicans* strain CA10 *in vitro*, this drug-resistant *strain* was selected as the study object and FLC was selected as the representative azole antifungal drug for use *in vivo* and synergistic mechanism experiments.

Bulk AMH and FLC (purity ≥98% by HPLC) were purchased from Dalian Meilun Biotech Co., Ltd. (Dalian, China). Stock solutions of AMH or FLC were prepared in distilled water to a final concentration of 2560 μg/mL. Stock solutions were stored at −20°C. *Galleria mellonella* was purchased from Huiyude Co., Ltd (Tianjin, China).

### 2.2 Determination of *in vitro* antifungal effects against planktonic cells

The antimicrobial activity of combinations of AMH with azole antifungal agents was assessed in a suspension assay by the checkerboard method in accordance with Clinical and Laboratory Standards Institute guidelines for yeasts (CLSI, 2020). Serial twofold dilutions of the drugs were prepared in RPMI1640. The final concentration of fungal suspension in the 96-well microplates was 1×10^3^ cells/mL. After adding the drugs and fungal suspension, the 96-well microplates were incubated at 35°C for 24 h, and then planktonic cell growth was observed. The MIC_50_ was the lowest concentration of drug that inhibited planktonic cell growth by 50%.

*In vitro* interactions between AMH and azole antifungal agents were analyzed by determining the fractional inhibitory concentration index (FICI): FICI=FIC_AMH_ +FIC_azole_ = (MIC of AMH in combination/MIC of AMH alone) + (MIC of azole in combination/MIC of azole alone). FICI ≤ 0.5 indicated synergy, FICI>4 denoted antagonism, and 0.5<FICI ≤ 4 was considered no interaction.

### 2.3 Determination of *in vivo* antifungal effects using the *G. mellonella* infection model

#### 2.3.1 Survival curve assay

*G. mellonella* is a mature insect infection model, that has been used to study the virulence of pathogenic fungi and screen new antifungal compounds (Serrano et al., 2023). In the initial stage of this study, in order to build an infection model of *G. mellonella*, we systematically evaluated the effects of different concentrations (1 × 10^7^ cells/mL, 1 × 10^8^ cells/mL, 5 × 10^8^ cells/mL, 1 × 10^9^ cells/mL) of *C. albicans* CA10 on the survival rate of *G. mellonella* (**Supplementary Figure S1**). Based on this detailed analysis result, we carefully selected concentration 5×10^8^ cells/mL as the key infection concentration in the follow-up experiments. To determine the antifungal effect of AMH+FLC *in vivo*, the survival rate of G. mellonella larvae was calculated. On the basis of the previous research experience of our team or other researchers, FLC and AMH doses of drugs 1.6 μg/larva and 3.2 μg/larva respectively, and a survival rate monitoring time of 4 days were used (Lafleur et al., 2013; Karaman et al., 2017; Li et al., 2019, 2023a). In brief, 80 healthy larvae of uniform weight (200 mg ± 10 mg) were randomly selected for this experiment. and divided into four groups: control group(PBS), FLC group (1.6 μg/larva), AMH group (3.2 μg/larva) and AMH+FLC group (3.2 μg/larva + 1.6 μg/larva). The body surfaces of the larvae were disinfected with medical-grade alcohol, and 10 μL of CA10 suspension (5 × 10^8^ cells/mL) was injected into the final right leg of each larva. The larvae were incubated in the dark at 35°C for 2 h after CA10 inoculation, and then 10 μL of drug(s) was injected into the final left leg of each larva. After treatment, the larvae were incubated in the dark at 35°C, and the number of dead larvae in each group was recorded every day for 4 days. Larvae that did not respond to repeated touches with metal tweezers were recorded as dead.

#### 2.3.2 Histopathological study

To observe the development of *C. albicans* infection in *G. mellonella* larvae and study the therapeutic effects of the different drug treatments on the larvae, histological studies were performed. The process of larval allocation and inoculation with the CA10 suspension was the same as above. Two days after drug treatment, one larva was randomly selected from each group in a blinded manner and prepared as 7-μm -thick frozen sections. The tissue sections were stained with periodic acid Schiff reagent (PAS) and observed under a 4.2 × 10 fluorescence microscope (Olympus FSX100, Japan). All assays were repeated on three separate occasions.

### 2.4 Synergistic mechanism analysis

#### 2.4.1 Rh6G efflux assay

Changes in fungal efflux pump activity are a common mechanism of fungal resistance, which has spurred growing interest in the effects of new antifungal compounds on efflux pump activity. In this study, the rhodamine 6G (Rh6G, Sigma-Aldrich, USA) tracer method was used to determine whether AMH interferes with efflux pump activity in drug-resistant *C. albicans*. CA10 cells (1 × 10^5^ cells/mL) were oscillated in YPD medium at 200 rpm and 35°C for 17-18 h. Next, the CA10 cells were collected, washed three times with glucose-free PBS, and suspended at a concentration of 1 × 10^7^ cells/mL.Rh6G was added to the CA10 suspension at a final concentration of 10 μM, and oscillation of the CA10 suspension was continued in the dark for 50 min. To stop the absorption of Rh6G, the cells were placed in an ice bath for 10 min. The cells were then harvested and re-suspended in glucose-PBS (5%). AMH was added to obtain a final concentration of 4 μg/mL, and an untreated group was set up as a control group. Then, the mean fluorescence intensity (MFI) of Rh6G was measured by flow cytometry (FACS Calibur, BD Biosciences, USA) every 10 min (excitation wavelength: 488 nm, emission wavelength: 530 nm).

#### 2.4.2 Biofilm assay

The effects of the drug combinations on established biofilms were tested in 96-well microplates as described previously (Li et al., 2019). Briefly, drug-resistant *C. albicans* CA10 cells (5 × 10^6^ cells/mL) were cultured at 37°C in 96-well microplates containing RPMI 1640 for 90 min or 24 h. After incubation, planktonic cells were gently removed, and each well was rinsed thrice with sterile PBS. Then, serial twofold dilutions of the drugs were prepared in RPMI1640 and added to the microplates containing biofilms. The 96-well microplates were then incubated again at 35°C for 48 h. Biofilms without drug treatment served as the control. The sessile MIC (SMIC) was determined as the lowest drug concentration that inhibited over 80% of biofilms compared with the control. The FICI model described in section 2.2 was used to evaluate the synergistic effect of AMH + FLC on biofilms.

#### 2.4.3 Extracellular phospholipase activity assay

We used egg yolk agar plates to assess the effects of AMH and/or FLC on the extracellular phospholipase activity of drug-resistant *C. albicans* (Zhang et al., 2020). CA10 cells (1 × 10^6^ cells/mL) were treated with no drugs, AMH (4 μg/mL), FLC (0.25 μg/mL), or AMH (4 μg/mL) + FLC (0.25 μg/mL) and in cubated at 35°C for 24 h. Next, 10 μL of each CA10 suspension was inoculated on egg yolk agar plates, which were then incubated at 35°C for 72 h. The diameter of the CA10 colony and the diameter of the precipitating band on the plates were then measured. The Pz value representing extracellular phospholipase activity was calculated as Pz = colony diameter/(colony diameter + precipitation zone diameter). According to the Pz value, extracellular phospholipase activity was classified into five categories: Pz ≤ 0.69, very high; 0.70≤Pz ≤ 0.79, high; 0.80≤Pz ≤ 0.89, low; 0.90≤Pz ≤ 0.99, very low; and Pz=1 negative (Mendonca et al., 2022).

### 2.5 Statistical analysis

All experiments were independently repeated three times. GraphPad Prism (version 7.3.0, GraphPad, La Jolla, CA, www.graphpad.com) and IBM SPSS Statistics (version 22, SPSS, Chicago, IL) were used for charting and statistical analysis. The survival assay was analyzed by the Kaplan–Meier method. For all other assays, the statistical significance of differences between the treated and control groups was analyzed by Student’s t-test; *p* < 0.05 was considered statistically significant.

## 3 Results

### 3.1 Combinations of AMH with azole antifungal drugs have synergistic effects on planktonic cells of drug-resistant *C. albicans in vitro*

The antifungal effects of AMH alone against drug-susceptible and drug-resistant *C. albican*s strains were limited (MIC, 32-512 μg/mL). However, when drug-resistant *C. albicans* strains (CA10 and CA16) were treated with AMH combined with azole antifungal drugs (FLC, itraconazole, and voriconazole), synergistic effects were observed (FICI=0.01-0.13), which demonstrated that AMH significantly increased the susceptibility of drug-resistant *C. albicans* to azole antifungal drugs (**Table 1**). We also tested the synergistic effects of these combinations on eight drug-susceptible and drug-resistant non-*C. albicans* strains (*C. glabrata* and *C. krusei*). No synergistic effects of AMH combined with azole antifungal drugs were observed for the tested *C. glabrata* and *C. krusei* strains, with FICI=1.25-2.00. These results showed that the synergistic effects of combinations of AMH with azole antifungal drugs were unique to *C. albicans*.

**Table 1:** *In vitro* interactions of AMH and azole antifungal drugs against the planktonic cells of Candida species.

Drugs	Strains^a^	MIC (μg/mL) ^b^				FICI model ^c^	
		MIC_azole_	C_azole_	MIC_AMH_	C_AMH_	FICI	Interpretation of interaction
FLC+AMH	CA4 (S)	0.5	0.0625	64	64	1.13	no interaction
	CA8 (S)	0.5	0.125	32	32	1.25	no interaction
	CA10 (R)	>512	0.25	512	4	0.01	synergy
	CA16 (R)	>512	2	64	8	0.13	synergy
	CG1 (S)	4	4	1024	1024	2.00	no interaction
	CG8 (S)	8	8	1024	1024	2.00	no interaction
	CG2 (R)	128	128	>1024	>1024	2.00	no interaction
	CG3 (R)	64	64	>1024	>1024	2.00	no interaction
	CK2 (S)	>4	4	1024	1024	2.00	no interaction
	CK3 (S)	>4	4	1024	1024	2.00	no interaction
	CK9 (R)	64	64	>1024	1024	2.00	no interaction
	CK10 (R)	64	64	>1024	>1024	2.00	no interaction
ITR+AMH	CA4 (S)	0.0625	0.0625	256	128	1.50	no interaction
	CA8 (S)	0.25	0.125	32	2	0.56	no interaction
	CA10 (R)	>512	0.0625	512	4	0.01	synergy
	CA16 (R)	>512	0.0625	256	8	0.03	synergy
	CG1 (S)	1	0.5	>1024	>1024	1.50	no interaction
	CG8 (S)	1	1	>1024	1024	2.00	no interaction
	CG2 (R)	128	128	>1024	>1024	2.00	no interaction
	CG3 (R)	128	128	>1024	>1024	2.00	no interaction
	CK2 (S)	>1	1	1024	1024	2.00	no interaction
	CK3 (S)	1	1	1024	1024	2.00	no interaction
	CK9 (R)	128	128	>1024	>1024	2.00	no interaction
	CK10 (R)	128	128	>1024	>1024	2.00	no interaction
VRC+AMH	CA4 (S)	0.0313	0.0313	256	128	1.50	no interaction
	CA8 (S)	0.0625	0.0313	32	16	1.00	no interaction
	CA10 (R)	512	0.0625	512	8	0.02	synergy
	CA16 (R)	512	1	256	8	0.03	synergy
	CG1 (S)	0.125	0.125	>1024	>1024	2.00	no interaction
	CG8 (S)	1	0.25	>1024	1024	1.25	no interaction
	CG2 (R)	4	4	>1024	>1024	2.00	no interaction
	CG3 (R)	2	2	>1024	>1024	2.00	no interaction
	CK2 (S)	1	1	1024	1024	2.00	no interaction
	CK3 (S)	0.5	0.5	1024	1024	2.00	no interaction
	CK9 (R)	2	2	>1024	>1024	2.00	no interaction
	CK10 (R)	4	4	>1024	>1024	2.00	no interaction

a S indicates that the strain is susceptible to azoles, and R indicates that the strain is resistant to azoles.

b FLC, fluconazole; ITR; VOR; AMH; MIC_azole_, the MIC of azole antifungal drugs when used alone; C_azole_, the MIC of azole antifungal drugs when used in combination with AMH; MIC_AMH_, the MIC of AMH when used alone; C_AMH_, the MIC of AMH when used in combination with azole antifungal drugs.

c FICI ≤ 0.5: synergy; FICI > 4.0: antagonism; 0.5 < FICI ≤ 4.0: no interaction.

### 3.2 AMH + FLC has a good therapeutic effect *in vivo* in infected *G. mellonella*

**Figure 1:**
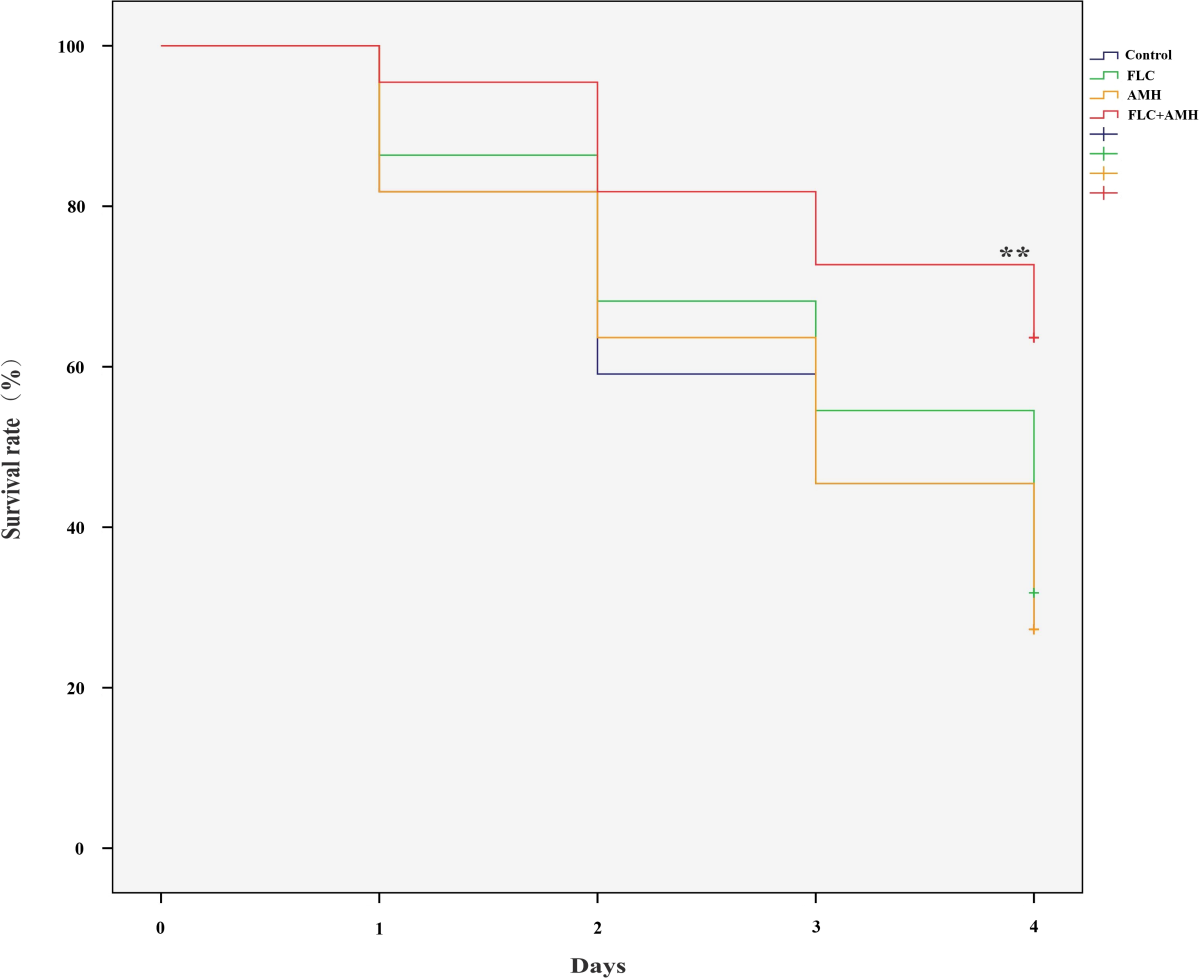
AMH+FLC synergistically prolonged the survival rate of *G. mellonella* infected with drug-resistant *C. albicans*. Larvae were infected with 5 × 10^8^ cells/mL of *C. albicans* CA10 and treated with PBS (control), AMH (3.2 μg/larva), FLC (1.6 μg/larva), or AMH + FLC (3.2 μg/larva+1.6 μg/larva) after 2 h of infection. **p<0.05 when compared with the control or the drug alone groups.

The survival rates of the four groups of larvae infected with CA10 over 4 days are shown in **Figure 1**. Compared with the control group, a significant improvement in the survival rate was observed only in the AMH+FLC group (*p* < 0.01). AMH+FLC treatment significantly prolonged the survival time and improved the survival rate of infected *G. mellonella* larvae compared with no treatment or treatment with either drug alone.

**Figure 2:**
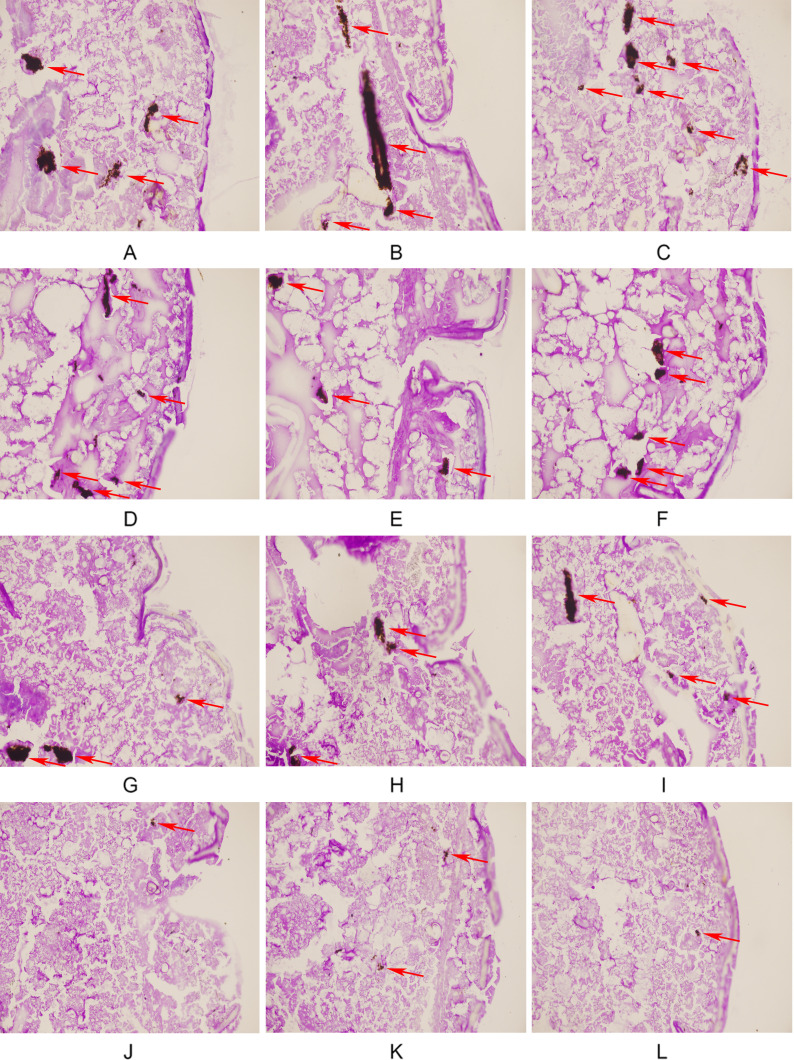
AMH+FLC synergistically reduced tissue invasion of *G. mellonella* infected with drug-resistant *C. albicans.* **(A-C)** Infected group, **(D-F)** FLC alone group, **(G-I)** AMH alone group, **(J-L)** AMH+FLC group. The infected area of *C. albicans* CA 10 is indicated by a red arrow.

**Figure 2** illustrates the tissue damage in the four groups of larvae. In the control group, a large number of black fungal masses and areas of tissue infiltration were observed, indicating a large fungal burden in the larvae and severe tissue damage caused by the fungus. Compared with the control group, the area and number of fungal masses were slightly lower in the FLC group and the AMH group, whereas the area and number of fungal masses were significantly lower in AMH+FLC group. In the AMH+FLC group, only small, spot-like clumps scattered in larval tissue were observed. The histological observations were consistent with the survival rates, and both demonstrated the good efficacy of the drug combination for treating CA10*-*infected *G. mellonella*.

### 3.3 Synergistic mechanisms

#### 3.3.1 AMH inhibits efflux pump activity in drug-resistant *C. albicans*

**Figure 3:**
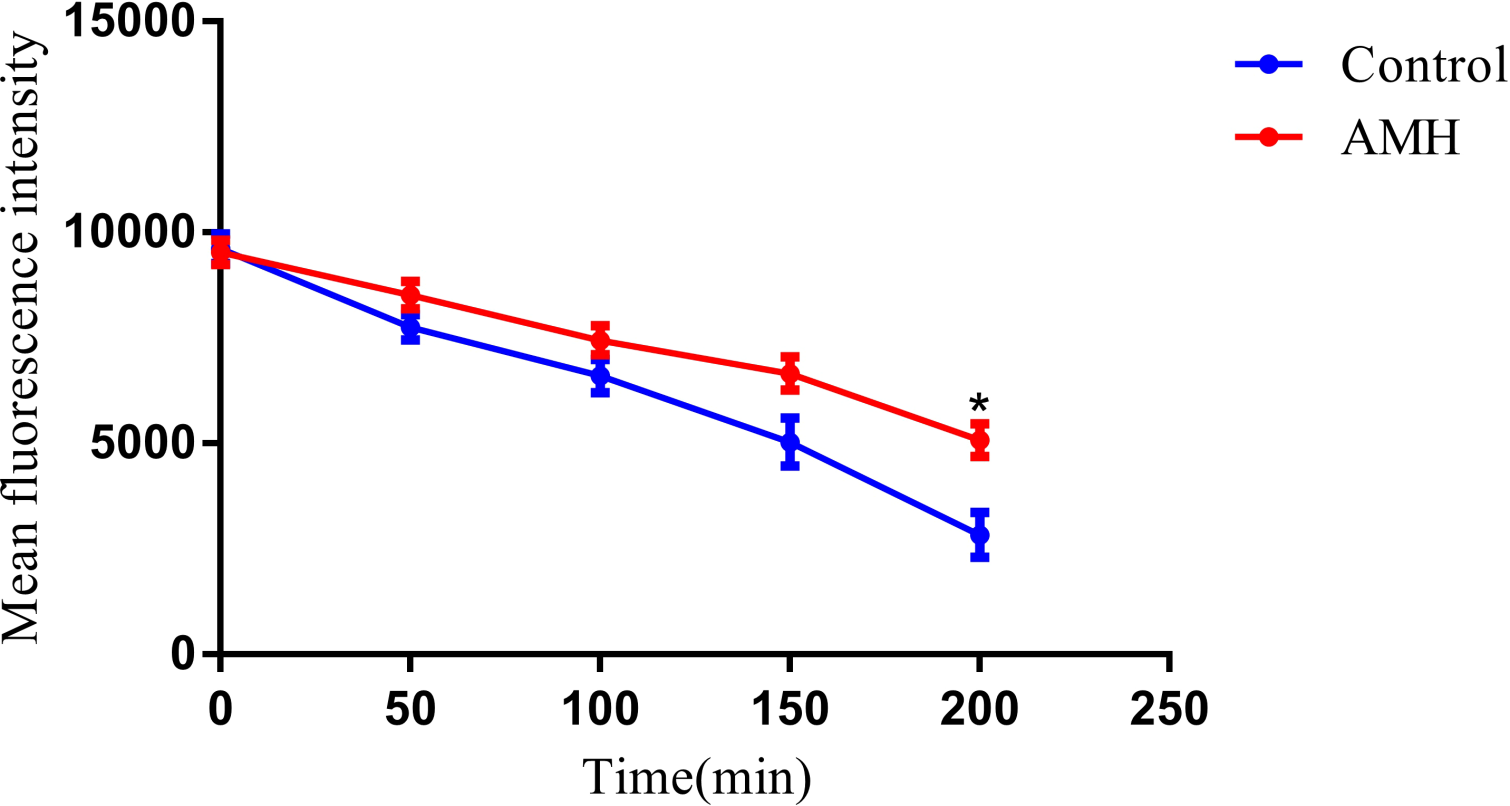
AMH inhibited the efflux of Rh6G in drug-resistant *C. albicans*. *p<0.05 when compared with the control or the drug alone groups.

In *C. albicans*, both azole antifungal drugs and the fluorescent dye Rh6G are substrates for efflux pumps on the cell membrane (Maesaki et al., 1999). We therefore used Rh6G to study the effect of AMH on efflux pump activity, The MFI of Rh6G was used as an indicator of efflux pump activity in CA10 cells (**Figure 3**). Over time increased, the MFI continued to decrease in both the control group and the AMH group, indicating an increase in the efflux of Rh6G. However, efflux activity was always significantly lower in the AMH group than in the control group, indicating that AMH significantly inhibited the efflux of Rh6G in CA10 cells (*p* < 0.05). This result suggests that the synergistic antifungal mechanism of the combination of AMH with azole antifungal drugs in drug-resistant *C. albicans* is related to the inhibition of drug efflux pump activity on the cell membrane.

#### 3.3.2 AMH + FLC has synergistic effects against early biofilms of drug-resistant *C. albicans*

Biofilm formation is an important virulence factor of *C. albicans and* is also an important mechanism of drug resistance. Biofilm formation by *C. albicans* leads to high levels of resistance against most antifungal agents (Hu et al., 2017). We therefore examined the antifungal effects of combinations of AMH with other drugs on resistant *C. albicans* biofilms. As shown in **Table 2**, the drug combinations inhibited drug-resistant *C. albicans* biofilms that were preformed for 90 min but not biofilms that were preformed for 24 h, indicating that AMH+FLC synergistically inhibited early biofilms of drug-resistant *C. albicans*.

**Table 2:** *In vitro* interactions of AMH and FLC against biofilms (90 min or 24 h) of resistant *C. albicans*.

Times	Strains ^a^	SMIC (μg/mL) ^b^				FICI model ^c^	
		MIC_FLC_	C_FLC_	MIC_AMH_	C_AMH_	FICI	Interpretation of interaction
90 min	CA10 (R)	>512	8	>512	8	0.0313	synergy
24 h	CA10 (R)	>512	>512	>512	>512	2.000	no interaction

a R indicates that the strain is resistant to azoles.

b SMIC: sessile minimum inhibitory concentration; SMIC_FLC_ and SMIC_AMH_: the SMICs when drugs used alone; C_FLC_ and C_AMH_: the SMICs when drugs used in combination.

c FICI ≤ 0.5: synergism; FICI > 4.0: antagonism; 0.5 < FICI ≤ 4.0: no interaction.

#### 3.3.3 AMH + FLC reduces the extracellular phospholipase activity of drug-resistant *C. albicans*

The extracellular phospholipase activity of drug-resistant *C. albicans* treated with different drugs is shown in **Table 3**. The Pz value of the control group, FLC group, and AMH groupwere0.67 ± 0.02, 0.64 ± 0.05, and 0.73 ± 0.02 respectively, demonstrating very high or high phospholipase activity. The Pz value of the AMH+ FLC group was 0.87 ± 0.06, much higher than those of the other three groups, indicating that AMH + FLC significantly inhibited the extracellular phospholipase activity of CA10, reducing its virulence. These results suggest that the synergistic antifungal mechanism of AMH+FLC involves inhibition of extracellular phospholipase activity in drug-resistant *C. albicans*.

**Table 3:** Extracellular phospholipase activity of CA10.

	Colony diameter (cm)	Colony diameter + precipitation zone diameter(cm)	Pz	Average±standard deviation of Pz	Phospholipase activity
Control	1.6	2.3	0.696	0.67±0.02	Very high
	1.6	2.4	0.667		
	1.6	2.45	0.653		
FLC	1.65	2.4	0.688	0.64±0.05	Very high
	1.6	2.75	0.582		
	1.5	2.3	0.652		
AMH	1.9	2.65	0.727	0.73±0.02	High
	1.8	2.5	0.720		
	1.9	2.55	0.750		
AMH+FLC	1.85	2.05	0.902	0.87±0.06 ^a^	Low
	1.8	2.25	0.800		
	2.1	2.3	0.910		

a *P* < 0.05 compared to the control.

## 4 Discussion

Approximately 6.55 million acute fungal infections are reported annually across more than 80 nations worldwide, leading to a staggering 3.75 million fatalities (Denning, 2024). This mortality count doubles the previously projected toll, underscoring the severity and threat of fungal infections. In parallel with the increase in resistant fungal isolates, the immunocompromised population is growing, which poses additional challenges in the treatment and management of fungal infections. Efforts to address the growing problem of fungal resistance are focused on, discovering new antifungal compounds and novel therapeutic strategies. Combining antifungal drugs with sensitizers is a particularly advantageous antifungal therapeutic strategy. Fungal strains are unlikely to develop resistance to both antifungals and sensitizers because such resistance would require multiple mutations. In addition, combining drugs reduces the dose of each drug, shortens the duration of treatment, and reduces toxicity to the host (Cokol et al., 2011; Campbell et al., 2012; Lu et al., 2021; Kane and Carter, 2022). Among pathogenic fungi, *C. albicans* accounts for the majority of systemic fungal infections, and resistant *C. albicans* is a particularly problematic clinical issue (Pappas et al., 2018). Our team has identified several sensitizers of conventional antifungal drugs to address the problem of resistance in *C. albicans* (Li et al., 2019, 2023a, b, c).

Inspired by the activity of the combination of the antiviral drug ribavirin and FLC against resistant *C. albicans* (Zhang et al., 2020), we examined whether combinations of other antiviral drugs with antifungal drugs also have synergistic effects on resistant *C. albicans.* In this study, we evaluated the antifungal activity of AMH alone or in combination with azole antifungal drugs against *C. albicans*. *In vitro* studies in planktonic cells showed that combinations of AMH with azole antifungal drugs had strong synergistic effects on drug-resistant *C. albicans*. When the concentration of AMH was 4-8 μg/mL, the MIC_80_ of azole antifungal drugs against drug-resistant *C. albicans* decreased from ≥ 512 μg/mL to 0.0625-2 μg/mL, and the FICI was 0.01-0.13, far less than 0.5. For drug-susceptible *C. albicans* and all non-*C. albicans* strains, the FICI was 1.125-2.00, and there was no interaction of AMH with azole antifungal drugs.

AMH+FLC had synergistic effects on drug-resistant *C. albicans* not only *in vitro*, but also *in vivo*. Because *G. mellonella* is easy to raise and infect, it is an ideal infection model for screening and evaluating antifungal drugs *in vivo* (Torres et al., 2020). Compared with the control group (without drug treatment) and groups treated with a single drug (FLC group and AMH group), the survival rate of *G. mellonella* infected with drug-resistant *C. albicans* CA10 4 days after treatment was significantly higher in the AMH+FLC group (*p* < 0.01). In addition, histopathological studies showed that AMH+ FLC treatment significantly reduced the damage and fungal load of infected *G. mellonella*, demonstrating that AMH+FLC had good synergistic antifungal effects *in vivo*.

After fully confirming the synergistic effects of AMH+FLC on drug-resistant *C. albicans in vivo* and *in vitro*, we explored the synergistic mechanisms of this drug combination. Such studies are crucial for providing a theoretical basis for the development of new drugs for combined applications. We focused on biofilm formation, and extracellular phospholipase activity and efflux pump activity, as these three functions are closely related to the development of drug resistance in *C. albicans*. Resistance to antifungal drugs in *C. albicans* is often associated with overactivity of drug efflux pumps (Hu et al., 2017). Inhibiting the activity of drug efflux pumps can increase the intracellular concentration of antifungal drugs in *C. albicans*, which not only improves the sensitivity of *C. albicans* to antifungal drugs and delays the development of drug resistance, but also reduces the antifungal drug dose antifungal drugs and the occurrence of side effects (Guevara-Lora et al., 2022). Biofilm formation increases the aggressiveness and resistance of *C. albicans* in some environments (Hu et al., 2017). Specifically, cell aggregation in a biofilm can create a multi-layered structure that prevents, direct contact and penetration of antifungal drugs (Lara et al., 2020). Biofilms also provide a relatively independent living environment in which the cells within the biofilm are able to metabolize and excrete antifungal drugs more efficiently, thereby reducing the intracellular concentrations of antifungal drugs (Sharma et al., 2019). Therefore, treating drug-resistant *C. albicans* infections requires not only the careful, selection of appropriate antifungal drugs but also full consideration of the impact of biofilms. The protective effects of biofilms can be overcome through the combined application of antifungal drugs with different mechanisms or the use of adjuvant therapy. AMH + FLC effectively inhibited immature biofilms that were preformed for 90 min. During this early stage of biofilm formation, most cells are adhered and in the yeast or early germ tube formation stages. Unfortunately, the combination lacked efficacy against mature biofilms. From a clinical perspective, biofilms are typically fully mature at the onset of treatment, and this limitation of AMH + FLC necessitates further consideration. Extracellular phospholipase is an enzyme secreted by *C. albicans*, that plays an important role in pathogenesis, infection and drug resistance This enzyme can degrade extracellular phospholipids, thus destroying the integrity of biofilms; extracellular phospholipase can also help *C. albicans* invade host tissue cells and inhibit the phagocytosis function of host immune cells (Nailis et al., 2010). Thus, inhibiting the secretion of extracellular phospholipase can reduce the destruction of fungal biofilms and enhance the resistance of the host to infection. The studies of the synergistic mechanism of AMH+FLC in drug-resistant *C. albicans* showed that AMH inhibited efflux pump activity, and extracellular phospholipase activity. Collectively, these results indicate that the synergistic mechanisms of combinations of AMH with azole antifungal drugs are related to an increase in the intracellular azole antifungal drug concentration, and inhibition of early biofilms and extracellular phospholipase activity.

AMH was first approved in 1966 for the treatment of influenza A virus, and its anti-Parkinson’s effect was discovered in the 1970s. AMH is the only drug proven to be effective for treating levodopa-induced dyskinesia in people with Parkinson’s disease (Nakhai et al., 1991; Fox et al., 2018). In 2013, LaFleur et al. found that 2-adamantanamine, which has a structure similar to that of AMH, had an anti-*C. albicans* biofilm effect when combined with miconazole (Lafleur et al., 2013). However, the effects of combinations of AMH with azoles on resistant *Candida* spp. have not been studied. Our study demonstrates that the non-antifungal drug AMH has synergistic effects with FLC and enhances the antifungal effect of FLC on resistant *Candida* spp. In future work, we will attempt to structurally modify AMH to obtain superior azole sensitizers and investigate the performance of AMH+FLC in a mixed fungal-viral infection setting.

## 5 Conclusions

This study found that the combination of the antiviral drug AMH with FLC (as a representative conventional azole antifungal drug) had significant synergistic antifungal effects on drug-resistant *C. albicans in vitro* (FICI=0.01-0.13). *In vivo*, FLC+AMH significantly improved the survival rate and reduced the tissue fungal load of *C. albicans*-infected *G. mellonella*. The synergistic antifungal mechanisms of FLC+AMH against drug-resistant *C. albicans* may be related to inhibition of the efflux of intracellular azole antifungal drugs, early biofilms, and extracellular phospholipase activity. These findings suggest that combining AMH with azole antifungal drugs is a promising treatment to overcome drug-resistant *C. albicans*. However, further studies in mammals and humans are needed to verify the effectiveness and safety of these combinations.

## Data Availability

The raw data supporting the conclusions of this article will be made available by the authors, without undue reservation.
