# Supplementary figures and images for: Synergistic antifungal effects and mechanisms of amantadine hydrochloride combined with azole antifungal drugs on drug-resistant Candida albicans
Source: Front Cell Infect Microbiol. 2025 Feb 26;15:1455123. doi: 10.3389/fcimb.2025.1455123 (PMC11897512; doi:10.3389/fcimb.2025.1455123)

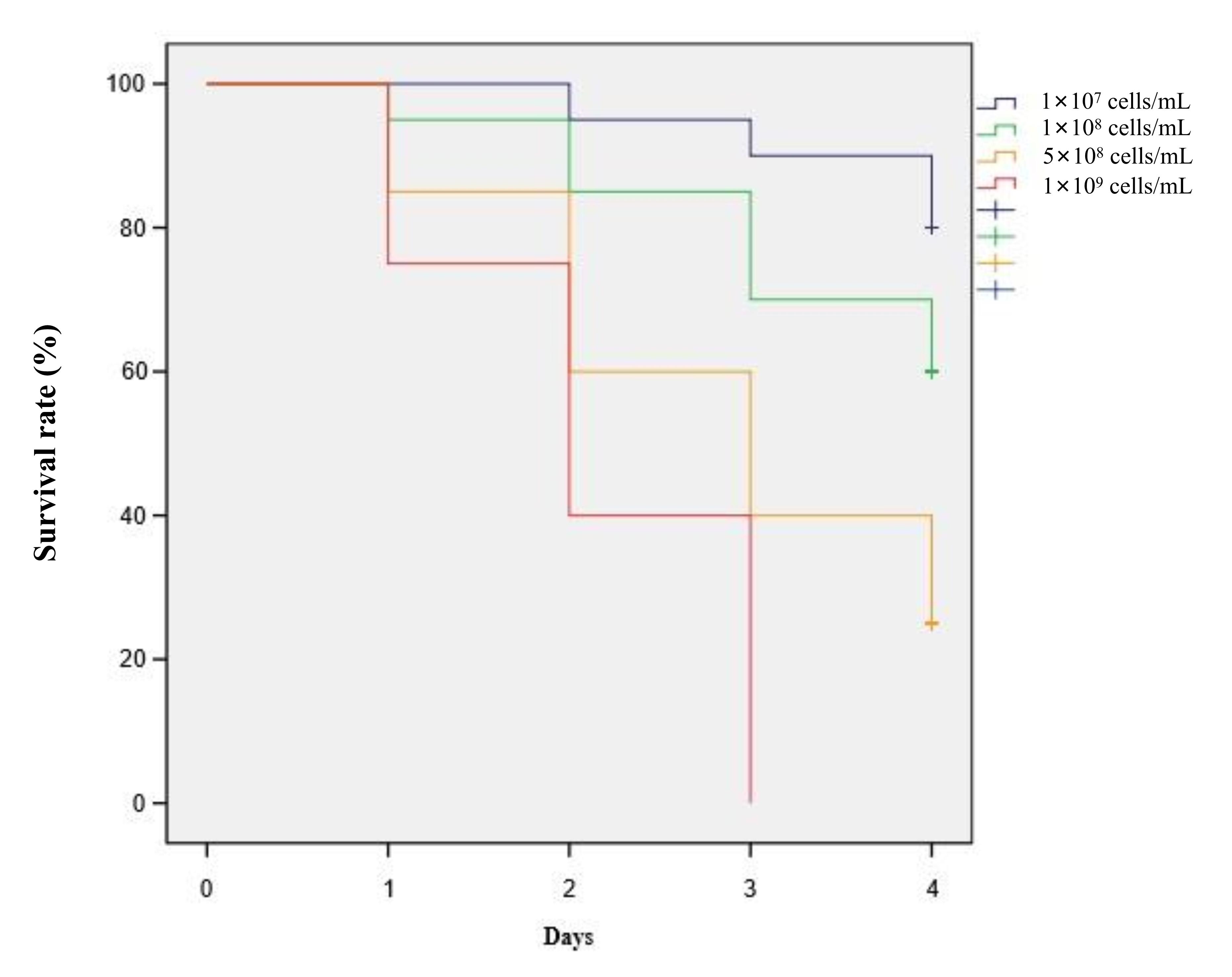

Supplement: Supplementary file 1 [file Image1.jpeg]
