# Supplementary material for: Synergistic antifungal effects and mechanisms of amantadine hydrochloride combined with azole antifungal drugs on drug-resistant Candida albicans
Source: Front Cell Infect Microbiol. 2025 Feb 26;15:1455123. doi: 10.3389/fcimb.2025.1455123 (PMC11897512; doi:10.3389/fcimb.2025.1455123)
Supplement: Supplementary file 2 [file DataSheet1.pdf]

**Table S1. MICs (μg/mL) of azoles antifungal drugs for all strains in this study**

| Strains [a] | Fluconazole [b] | Voriconazole [c] | Itraconazole |
|---|---|---|---|
| CA4 | 0.5 [S] | 0.0313 [S] | 0.0625 [#] |
| CA8 | 0.5 [S] | 0.0625 [S] | 0.25 [#] |
| CA10 | >512 [R] | 512 [R] | >512 [#] |
| CA16 | >512 [R] | 512 [R] | >512 [#] |
| CG1 | 4 [#] | <0.5[#] | 1 [#] |
| CG2 | 128 [R] | 4 [#] | 128 [#] |
| CG3 | 64 [R] | 2 [#] | 128 [#] |
| CG8 | 8 [#] | 1 [#] | 1 [#] |
| CK2 | >4 [#] | <0.5 [#] | <0.5 [#] |
| CK3 | >4 [#] | <0.5 [#] | <0.5 [#] |
| CK9 | 64 [#] | 64 [R] | >512 [#] |
| CK10 | 128 [#] | 4 [R] | >512 [#] |

[a] CA: Candida albicans; CG: Candida glabrata; CK: Candida krusei

b R: Resistant. According CLSI M60 (2020), the resistant breakpoint of fluconazole against Candida albicans and Candida glabrata is ≥8 μg/mL and ≥64 μg/mL, respectively. S: Susceptible. The susceptible breakpoint of fluconazole against Candida albicans is ≤2 μg/mL.

c R: Resistant. The resistant breakpoint of voriconazole against Candida albicans and Candida krusei is ≥1 μg/mL and ≥2 μg/mL, respectively. S: Susceptible. The susceptible breakpoint of voriconazole against Candida albicans is ≤0.12 μg/mL.

# breakpoints have not been reported.
